# Liquid fuel oil produced from plastic based medical wastes by thermal cracking

**DOI:** 10.1038/s41598-021-96424-2

**Published:** 2021-08-23

**Authors:** Shahriar Bin Rasul, Uday Som, Md. Shameem Hossain, Md. Wasikur Rahman

**Affiliations:** 1 Department of Chemical Engineering, Jashore University of Science and Technology, Jashore, 7408 Bangladesh; 2 Department of Energy Science and Engineering, Khulna University of Engineering and Technology, Khulna, 9203 Bangladesh

**Keywords:** Environmental sciences, Energy science and technology

## Abstract

The present work is an effort to produce liquid fuel oil from plastic based medical wastes through thermal cracking process under oxidizing conditions. The mixed plastics from medical wastes were considered as a feedstock, shredded into small pieces and heated at 773 ± 10 K for 40 min with a heating rate of 20 K/min in a batch reactor for thermal cracking process. The feedstock was characterized by proximate and ultimate analysis along with thermogravimetric investigation. Moreover, chemical compositions of the liquid fuel oil were examined by FTIR and GC–MS spectroscopy. The properties of liquid product were also examined and compared to the commercial fuel oil. The average yield of brownish and sticky liquid fuel was obtained to be 52 wt% and the gross calorific value of the liquid was found 41.32 MJ/kg which is comparable to that of commercial diesel. FTIR spectrum showed characteristic absorption bands of C–H and =CH_2_ groups indicating presence of alkane and alkene compounds. GC–MS study demonstrated the chemical constituents of the liquid product that is mostly aliphatic compounds of mainly alkanes (16.28%), alkenes (10.67%), alcohols (14.65%) and ester groups (10.38%) including iso-phthalate (40.02%) as a predominant product. This experiment concludes that the liquid oil derived from thermal cracking of mixed plastics comprised of a composite mixture of organic components. A significant amount of non-degraded constituents like plasticizers, precursors, etc. remained in the product having some economic values with human health and environmental impacts during burning has been addressed in the current issue.

At present, we face an energy crisis and environmental dilemma due to rapid increase of population and industrialization worldwide. Huge quantity of solid wastes has been invariably refused every day from various potential sources like household, industry, municipal areas, medical or clinics, etc. Nowadays pollution of plastic has come to remain an indispensable component of recent social life^1^. These wastes are often subtly transformed into renewable energy by diligently following appropriate methods that might be used rather than the fossil fuel. Modest recovery of alternative fuel and reducing plastic waste, the state-of-the-art technologies are developed day by day and this is often suitable for cost-effective and as of the environmental viewpoint^2^. Plastic materials, for remarkable instance, include a progressively growing proportion of the industrial and municipal wastes. The annual plastic consumption of the world has augmented around twenty times from five million tons in 1950 to approximately a hundred million tons within the most prior time. Unevenly around 5% plastics are being exclusively recycled and hereafter constant attempts are being articulated to grow the recycling of waste-plastic vigilantly^3^. Mixed waste plastics devise significant calorific value of fluctuating overall around 30–40 MJ/kg can be efficiently utilized as an alternate source of renewable energy^4^.

Medical waste is categorized as both solid and liquid states. Plastic based medical wastes are the solid sort of medical wastes that includes vials, syringes, infusion set, infusion pipes, covers, medicine containers, packets, etc.^5^. Due to hazardous and infectious characteristics of medical waste, it retains severe threat for the environment and needs exact treatment and managing prior to final disposal^6^. Medical wastes are proficient in causing diseases and disorders to people through either direct contact or ultimate contaminating environmental route of air, water and soil. Consequently, if the medical wastes are not carefully handled, the society and the surrounding environment possess at risk^7^. The medical wastes are generally managed through various steps that include segregation, disinfection, storage, transportation, collection and disposal in landfills or recycling. Numerous approaches of recycling, for instance, biological, mechanical, thermo-chemical recycling etc. are followed in medical waste management^8^. Mechanical recycling is often actual process but it is limited to thermoplastics, contamination level, homogeneity of the kinds and color similarity^9^. The foremost remarkable plastic waste management method is chemical recycling. Incineration and pyrolysis are usually employed to obtain biofuel from plastic materials. Nonetheless, pyrolysis is the key process to alter plastics into liquid oil effectively that comprises heat^10^.

Incineration is a crucial procedure from which hydrocarbons are altered into combustion products; whereas, pyrolysis is often employed to change them into inferior hydrocarbons can be employed as biofuel and different materials having economic values. An important magnitude of hazardous pollutants, like HCl, furans, dioxins and heavy metals including Hg, Cd and Pb, are formed from the incineration of medical wastes^11^. Pyrolysis is a process of thermal cracking that occurred in the absence of oxygen. By pyrolysis method, the organic constituents of the decomposable material yield various products of liquid oil, syngas and char as potential sources of energy^12^. The composition of the products depends upon process parameters just as temperatures of the consequent steps of condensation^13^. Various research outcomes reported in the literature that pyrolysis method is usually operated at temperature ranges of 623 and1173 K for 15 to 120 min^14^. Products obtained by pyrolysis of waste plastics lay on the characteristics of plastics, duration, feeding plan, reactor type, condensation arrangement and temperatures employed^15^. The excellence of liquids resulting from the pyrolysis of waste plastics extensively depends on the procedure and therefore the load^16^. Now, the reclamation fuel gas is castoff inside the plant as supplementary fuel for the source of energy to the gas-triggered boiler^17^. The physico-chemical performance of plastics for the amount of thermal pyrolysis acting a crucial role in reactor design^18^. Various reactors are set up and utilized, for example, batch or semi-batch, fluidized bed, screw kiln, spouted bed, microwave and fixed bed^19^. Semi-batch, batch and fixed bed devices are cast off by many investigators as a result of its modest design and informal procedure.

In general, the waste plastics are being recycled especially polypropylene (PP), low density polyethylene (LDPE) and high density polyethylene (HDPE) and polyvinyl chloride (PVC) based plastics. Due to hazardous and infectious contents in medical plastics, these need to be incinerated. However, burning plastic releases toxins, like furans and dioxins, persistent organic pollutants (POPs), polycyclic aromatic hydrocarbons (PAHs), etc. results in air and environmental pollution^20^. Emission of polychlorinated dioxins and dibenzofurans like toxins was quantified from medical waste incinerators (MWI) in China and the residents nearby the MWI are at high health risk^21^. In fact, the direct combustion of organo-chloride compounds like PVC is the main cause of those toxic releases^22,23^. Mechanical recycling is one of the alternative eco-friendly options for PVC based plastic waste management but it is necessary to separate out from other types of plastics^24^. In case of infectious hazardous medical wastes, the separation process of PVC materials is at risk for human health. It has been reported that dehydrochlorination is a predominant process for decomposition of PVC at low temperature^25^ and no harmful dioxin or furan compounds were found in pyrolytic yield from PVC and mixed plastics which was carried out at higher temperature^26,27^.

Catalytic degradation of plastic waste polymers and compared the results to catalytic and non-catalytic degradation^28^, have examined the thermal cracking of plastic waste^29^. A study on thermal decomposition of LDPE operated at 623 K in oxidizing atmosphere showed that linear aliphatic compounds mostly low carbon alcohols and carboxylic acids were obtained^30^. Butler et al. showed the pyrolysis of plastic syringe as medical wastes to produce valuable fuel oil^31^. Therefore, energy recovery from medical-based plastic wastes is one of the alternative solutions. A few pieces of scientific literature were reported on the thermal treatment of the medical wastes in oxygen free atmosphere and the characteristics of the liquid fuel oil^32^. Nonetheless, no one investigated the current issues in details as of transformation of medical based mixed plastic-wastes into energy through oxidative thermal degradation.

In medical sector, a huge amount of plastics based disposable medical supplies, made of HDPE, PP, PS, PVC etc., are discarded, which are a great source of mixed engineered plastics^33^. Since the natures of such medical grade plastics are specialized due to its high heat resistance, more durable, impact strength and highly stable under UV irradiation, there may have impact on the yield of liquid fraction from thermal treatment. Likewise, these outstanding properties of medical-grade plastics contain various additives, plasticizers, slip and curing agent etc. that may influence the characteristics of the thermally cracking fuel oil^34^. Moreover, disposable medical-based plastic wastes are disposed of every day from the hospitals, clinics and the diagnostic centres, which perform as a vector of spreading of communication diseases. Due to application of high temperature (> 573 K) in thermal treatment all germs even viruses are destroyed. A recent study shows that high temperature pyrolysis technique is one of the alternative technology in-practice to deal with the current pandemic COVID-19 based waste management^35^. So, the thermal treatment of mixed plastic wastes either the pyrolysis or the oxidative thermal cracking process could be a potential alternative medical waste management along with a resource recovery option. In Bangladesh, incinerator is used for managing all sort of medical wastes in few big cities whereas in urban/peri-urban areas the decomposable wastes are dumped or buried at the landfill site and the plastic wastes go to the recycler which is alarming for public health^36^.

Under these circumstances, the present work is an effort to produce liquid fuel oil from plastic based mixed medical wastes through thermal cracking in a batch reactor under oxidizing atmosphere and its characterization (Fig. 1). In order to ensure the quality of the derived fuel oil, the properties of the fuel has been studied and compared to the commercial fuels. Besides, the chemical characteristics has been undertaken to find out the nature and types of degraded constituents from the waste plastics and whether any harmful volatile compounds exist that have environmental and health effects. This work additionally contributes in developing proper management of plastic based medical wastes in order to reduce harmful environmental emissions other than a potential feedstock for liquid fuels.

**Figure 1:**
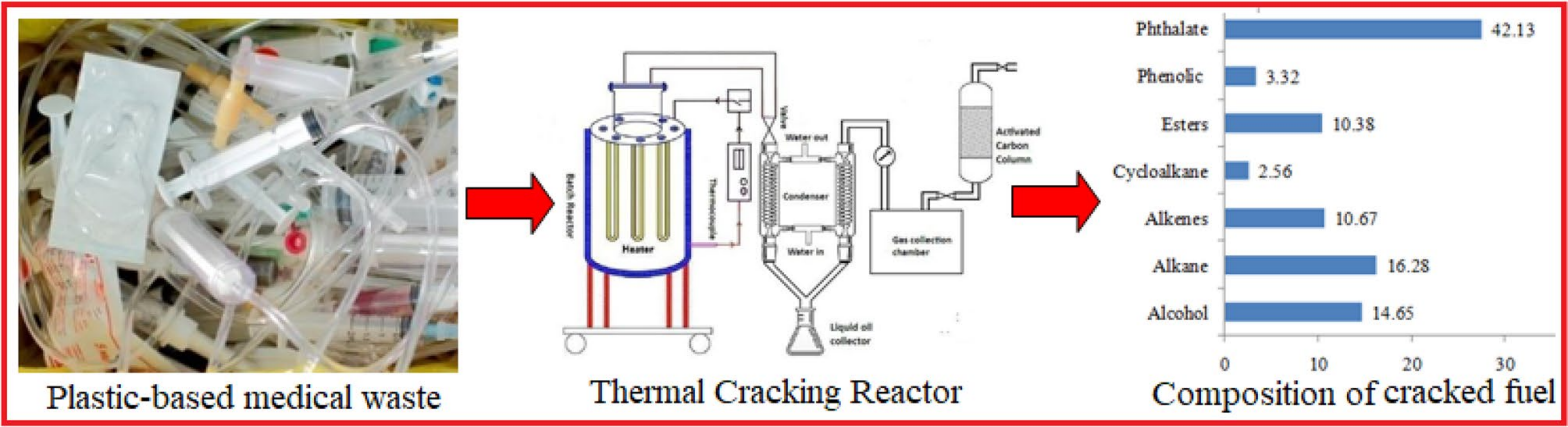
Graphical presentation of thermal cracking process for the production of liquid fuel oil from plastic based medical wastes and method of characterization.

## Materials and methods

### Feedstock

Plastic based medical wastes, mostly medical disposable supplies, were collected from the local hospitals and clinics of Bangladesh. This includes syringes, surgical gloves and infusion bags and sets that have been used as feed materials throughout the experiment. The composition of the medical supplies was identified from the specification of the manufacturers available in the literatures^33,37,38^. The different parts of the syringe, i.e., body, plunger and elastomeric piston seal are made of medical grade polyethylene [(C_2_H_4_)_n_], polypropylene [(C_3_H_6_)_n_] and polydimethylsiloxane [((CH_3_)_2_SiO)_n_; silicone rubber], respectively. The surgical gloves are made of synthetic rubber derived from nitrile butadiene rubber [(C_3_H_3_N)_n_·(C_4_H_6_)_m_]. Hard and soft both types of infusion bags were collected which were made of high density polyethylene [(C_2_H_4_)_n_] and medical grade plasticized PVC [(C_2_H_3_Cl)_n_], respectively. The infusion sets consist of various polymeric compounds in its various parts. The tubing and drip-counting chamber of the infusion set are made of medical grade PVC and polypropylene. The perforator and flow regulator of the infusion set are composed of plastic Acrylonitrile Butadiene Styrene copolymer (ABS) ([(C_3_H_3_N)_x_·(C_4_H_6_)_y_·(C_8_H_8_)_z_]), polyethylene and polystyrene [(C_8_H_8_)_n_], respectively. In addition, most of these medical grade plastics contain various additives like plasticizer, precursor, slip agent, curing agent, stabilizer, etc. to make them transparent, flexible, resistant to kinking and non-reactive to the fluids and medicines. So, the feedstock, selected for this experiment, is a composite and heterogeneous polymeric-additive compound. The collected plastic based medical wastes were cleaned successively by water, methanol and hydrochloric acid and eventually rinsed with distilled water and air-dried. This pre-cleaning approach was followed as a precaution for the researchers ensuring safe handling of the samples and avoiding communication disease or any pathogenic contamination. It is assumed that this pre-cleaning process does not necessarily important for large scale application of thermal cracking because all pathogens are killed at high temperatures. The washed materials were shredded to an average particle size of 1 cm and mixed thoroughly preparing as about 12 kg feedstock for a series of thermal cracking experiments. The size of the feedstock was predetermined based on the previous work of the research group^2^.

### Characterization of raw materials

Proximate and ultimate analysis of the feedstock was administered by a muffle furnace (Barnstead) and an elemental (CHNS) analyzer (Vario MICRO, Model EA 1108), respectively. The high calorific value (HCV) of the feedstock was measured by a Bomb Calorimeter. Degradation characteristics of the plastic based mixed medical wastes were measured by Thermogravimetric Analyzer (TGA) using Perkin-Elmer TGA7 (Detector TGA-50H, Cell Al). The TGA experiment was investigated at three altered heating rates of 10, 20 and 30 K/min with constant flow of air (oxidizing environment) at a rate of 10 mL/min from room temperature to 1073 K.

### Experimental procedure

**Figure 2:**
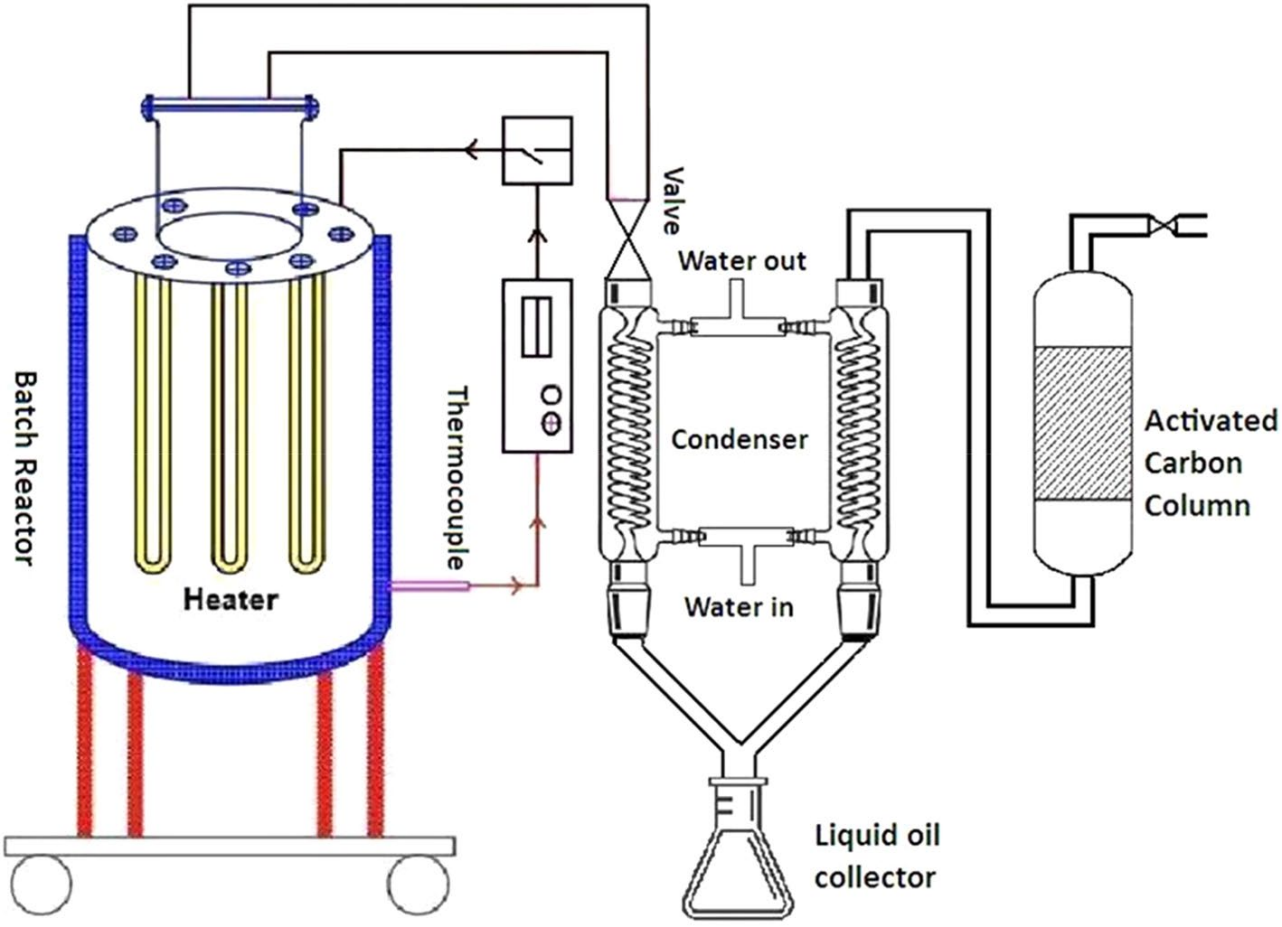
Experimental set up of thermal cracking of plastic based medical wastes.

The apparatus used in this experiment was a batch reactor which was cylindrical in shape, made of carbon steel, having 45.7 cm and 25.4 cm inner depth and diameter, respectively. Four electrical heating coils with a power of 500 W each were used as a source of heating energy, placed inside the reactor. A type K thermocouple connected with a temperature recorder was used for measuring temperature of the inside reactor. At the top of the reactor, a tubing system was connected with a valve through which the volatile fractions were flown in two successive condensers. The condensed product was stored in the oil collector and the residual non-condensable fraction was allowed to flow through a column of activated carbon and was exhausted to the air by controlling the exit valve. A schematic illustration of the thermal cracking experiment is shown in the Fig. 2.

The preliminary experiment was intended to discover the impact of temperature on the yield of fuel oil and char from thermal cracking of plastic based medical wastes. To this effect, the thermal cracking was carried out at four temperatures settings of 523 K, 623 K, 723 K and 773 K (± 10 K each) and a fixed amount of feedstock was loaded each time into the reactor. The thermal reaction was completed at a heating rate of 20 K/min under the normal oxidizing conditions for 25 to 40 min in different temperature settings. The derived liquid oil was collected after each reaction step and weighed. The char products, after each reaction step, were also collected by scooping out from the reactor and weighed. As described by a number of researchers^37,39^, the mass of the non-condensable fraction (M_g_) was determined from the material balance equation as follows:

$${\text{M}}_{{\text{g}}} = {\text{ M}}_{{\text{f}}} {-} \, \left( {{\text{M}}_{{\text{c}}} + {\text{ M}}_{{\text{o}}} } \right)$$

where M_f_ = Mass of feedstock considered for the thermal cracking, M_c_ = Mass of char product obtained from different temperature settings, M_o_ = Mass of liquid oil obtained from different temperature settings.

Due to the objective of this study, the non-condensable fraction was not collected for its characteristic investigation; however, creative works of current researchers of the related area are referred to understand its compositions. The liquid fraction obtained from the experiment repeated for three times at 773 ± 10 K was stored for measuring chemical features.

### Characterization methods of liquid fuel oil

To determine the physical properties (gross calorific value, density and flash point) of the derived liquid oil, the subsequent standard methods are followed: American Standard for Testing and Materials (ASTM), Density 4052 (ASTM: D4052), ASTM: D445-11, ASTM: D93, ASTM D97 and Oxygen-Bomb Calorimeter. Fourier Transform Infrared (FTIR) was employed to analyse surface properties of the sample using FTIR spectrophotometer (Model: IRAffinity-1S, Shimadzu Co., Japan). An analytical method of Gas Chromatography-Mass Spectroscopy (GC–MS) (GCMS-TQ8040) was carried out to identify the composition of the as-prepared liquid sample and works with a coupling of GC (column and injection temperatures 50 and 300 °C, respectively) and MS (acquisition time 3.5 to 36.5 min with an scan speed of 2000/sec).

## Results and discussions

### Elemental and proximate analysis of feedstock

The proximate analysis interprets that the feedstock contains 87.2% volatile matter, i.e., the bulk of the raw material is organic compounds which were burnt in the gaseous state. The fixed carbon in the feedstock is 7.8% that was burnt in the solid state. Higher volatile components quantified higher liquid product during the thermal cracking process. The volatile components and the high calorific value (HCV) are the crucial factors for the thermal treatment of plastic based medical wastes. It was found that 33.3 MJ of heat energy can be liberated from the combustion of 1 kg of medical wastes, indicated a good source of energy valorisation. The HCV value of the current study was found to be a little bit lower than those reported some other researchers as shown in Table 1, which might be due to the presence of some impurities, need to be further purified.

**Table 1:** Proximate and ultimate analysis of plastic based medical wastes.

References	Type of feedstock	Moisture	Volatile	Ash	Fixed carbon	H.C.V (MJ/kg)
**Proximate analysis (wt%)**						
Present work	PP, PE, PVC	0.82	87.2	4.17	7.81	33.3
Linbo et al.^20^	PP, PS	0.32	99.13	–	0.55	42.65
Singh et al.^28^	PE, PP, PS and PET	0.6	85.7	13.3	0.4	39.46

	Carbon (C)	Hydrogen (H)	Nitrogen (N)	Sulphur (S)	Oxygen (O)	Other
**Ultimate analysis (wt%)**						
Present work	72.56	11.17	3.82	0.227	–	12.22
Linbo et al.^20^	81.81	5.68	0.15	0.11	–	0.08
Singh et al.^28^	79.77	15.47	2.76	0.0	2.0	–

The ultimate analysis provides information about four elements as C = 72.6%, H = 11.2%, N = 3.8%, S = 0.23% and additional elements of 12.2% possibly O, Cl, Si, etc. present in the plastic wastes. The main compositions of medical plastic wastes used in this experiment were polyethylene (PE), polyvinyl chloride (PVC), polypropylene (PP), polystyrene (PS), acrylonitrile butadiene styrene copolymer (ABS), polydimethylsiloxane (PDMS) rubber and nitrile butadiene rubber (NBR), etc.^37,38^. All the polymeric materials are composed based on carbon and hydrogen skeleton. A small part of nitrogen comes from ABS and NBR. Chloride and silicon are assumed as additional elements (12.2%) in the ultimate analysis of PVC and PDMS. Oxygen is also presumed within 12.2% due to its presence in PDMS and various chemical additives (e.g., plasticizer, precursor and stabilizers, etc.) used in the processing of medical-grade plastics^40,41^. Sulphur may be found from sulphur containing antioxidant, which is used as a thermal stabilizer during the processing of polymeric materials^39^. The ultimate and proximate analyses of plastic medical wastes presented in Table 1 are compared to some other results reported elsewhere^20,42^.

### Characterization by TGA

**Figure 3:**
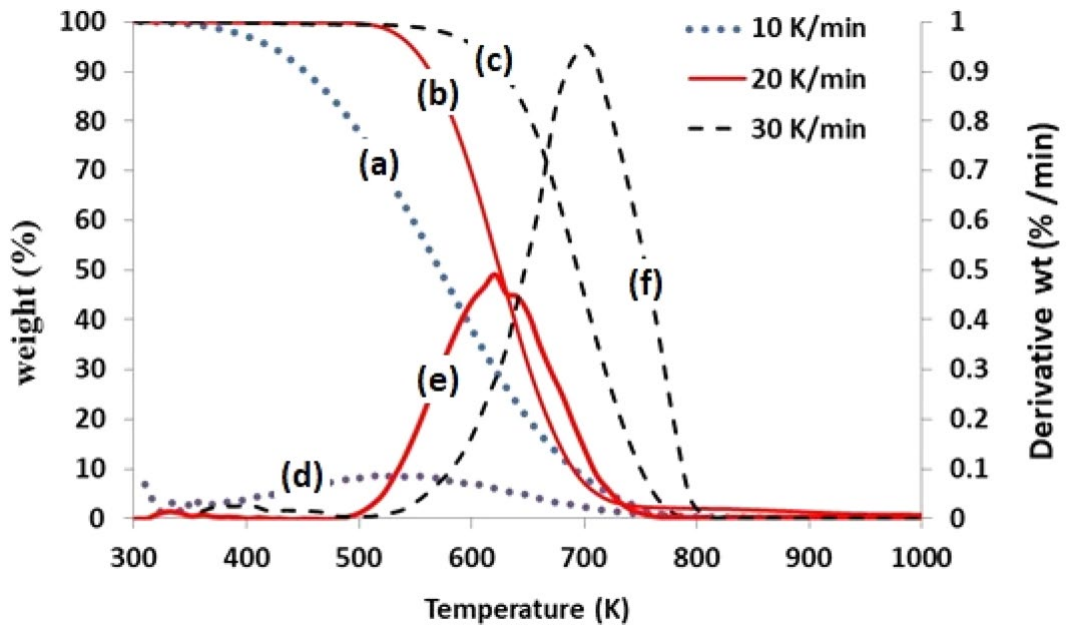
TG curves (**a**–**c**) and DTG peaks (**d**–**f**) of plastic based medical wastes at different heating rates.

The TGA result supports to understand the thermal stability of the feedstock and the behaviour of the degradation under controlled atmosphere like either in inert or in oxidizing environment. In this research, the plastic based mixed medical wastes (PE, PP, PVC, etc.) were considered for TGA at three heating rates of 10 K/min, 20 K/min and 30 K/min under oxidizing conditions. The Thermogravimetric (TG) curves (3a, 3b and 3c in Fig. 3) interpret the weight loss as a function of temperature at different heating rates corresponded to Differential Thermogravimetric (DTG) peaks (3d, 3e and 3f in Fig. 3) which construe the weight reduction of the feed sample with time. Figure 3 clearly indicates that both TG curves and DTG peaks moved to the elevated temperatures with increasing trial heating rates.

The DTG peaks also indicate wide degradation temperature spans which is due to mixed polymeric components in the plastic wastes^42^. The mixed plastics started decomposition from 360 K, 548 K and 555 K at the heating rates of 10 K/min, 20 K/min and 30 K/min, respectively under constant air flow. The degradation process was completed at 730 K, 740 K and 809 K with no residuals of significant level of char product. It was comprehended that degradation temperature was quite low at the minimum heating rate but it showed maximum ranges of temperature span. This TGA study revealed that the degradation temperatures at the heating rates was found significantly lower than those of individual virgin materials (like PE, PP, etc.) as reported by the researchers. This might be due to the presence of various additives, plasticizers, stabilizers, etc. in the plastic materials^42^. The degradation temperatures at which the weight loss became half of the feedstock were found to be 548 K, 621 K and 704 K corresponded to the successively increased heating rates. The DTG peak at 20 K/min heating rate (Fig. 3e) also showed two steps degradation at the temperatures of 621 K and 645 K results in possibly mixture of polymeric components in the plastic wastes. Based on the TGA data, finally, the liquid fuel oil was derived from the mixed plastic wastes by thermal cracking in the range of 500–773 K at a heating rate of 20 K/min under oxidizing environment.

### Effect of temperature on thermally degraded yield

**Figure 4:**
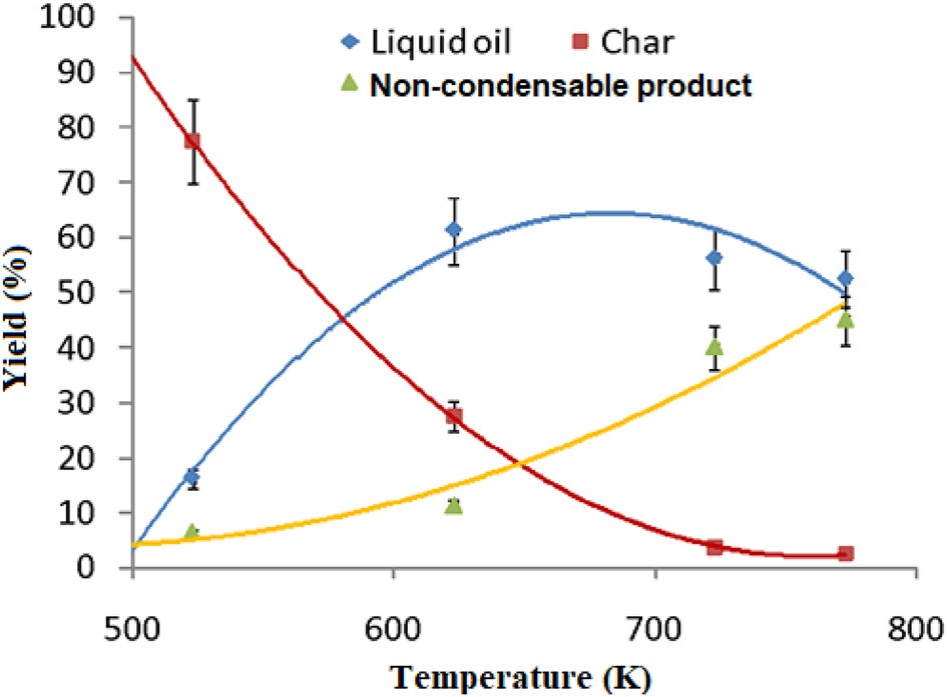
Effect of temperature of degraded yield of plastic based medical wastes.

In thermal cracking process, the degraded plastic materials are mainly char, condensate/liquid oil and a fraction of non-condensable product or gaseous fraction. The amounts of these degraded yields depend on the temperature applied during the thermal cracking. In this experiment, the effect of temperature was examined on the thermally degraded yields from plastic based mixed medical wastes as shown in Fig. 4. It was observed during the experiment that the maximum yield of liquid oil was found to be 61% with an optimum temperature of 623 K and the rate of yield was further decreased to 52% with the increasing in temperature at 773 K. Simultaneously, the yield of char was decreased successively and at the maximum temperature of 773 K, the amount of char became negligible. The calculated non-condensable fractions were found to be increased with temperature rises.

Dash et al.^37^ showed that 83.3 wt% of liquid oil was acquired from the pyrolysis of medical wastes (e.g., plastic syringe, a mixture of PP and PE). On the other hand, Hossain et al.^2^ showed that 39.89 wt% liquid yield was derived from the waste plastics (PP, PE, etc.) through pyrolysis process at 773 ± 10 K. Hence, there was a variation in product yields among the researchers in pyrolysis (in absence of oxygen) process. In this experiment, the maximum yields of liquid oil obtained through thermal cracking in oxidizing conditions do not differ significantly from the pyrolysis process. The obtained liquid product was brownish and sticky. It is depicted that thermal cracking of plastic materials depends on their distributions, range of temperature, composition of feedstock, types and dimensions of reactor, effectiveness of heat transfer from hot reactor surface to raw material, particle size of feedstock and residence time of vapor, etc. In the current experiment, the liquid oil derived from the maximum temperature setting at 773 ± 10 K was considered for chemical characterization because at this stage, the char production was negligible and the fraction of non-condensate was maximum value.

### Characterization of liquid fraction by FTIR

**Figure 5:**
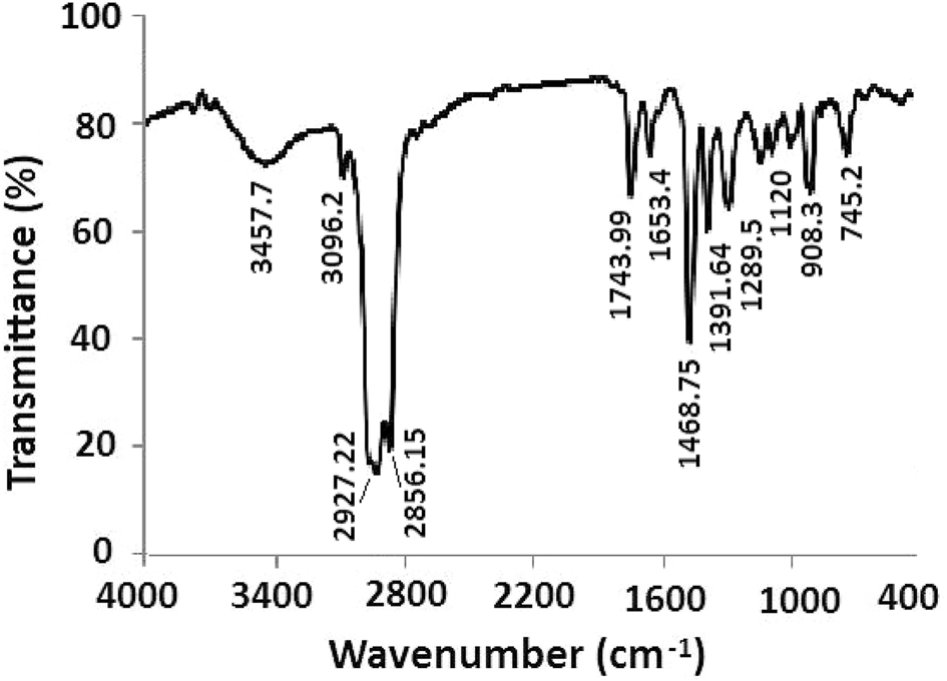
FTIR spectrum of liquid oil obtained from thermal degradation of plastic based medical wastes.

The purpose of FTIR analysis is the exploration of functional groups of organic compounds exists in the as prepared liquid-oil. The FTIR spectrum of derived liquid oil from the medical wastes is shown in Fig. 5. Strong absorption bands of stretching vibrations observed at 2927.22 cm^−1^for C–H and the bending pulsations of –CH_2_– group appeared at 1468.75 cm^–1^ for the alkane group. Similar bands for =C–H and C=C stretching vibrations were found at 3096.16 cm^−1^ and 1653.04 cm^−1^, respectively confirm the presence of alkene group. The broad and strong absorption peak for alcohol group (O–H bond) was found at 3457.7 cm^−1^and stretching vibration for alcoholic C–O bond was found at 1120 cm^−1^. The spectrum also showed the absorption peaks of stretching vibration for ester group at 1743.99 cm^−1^ for C=O bond and multiple peaks for –C–O stretching vibrations at 1050–1300 cm^–1^. FTIR analysis suggests that the organic compounds having the functional groups like alkanes, alkenes, alcohols, esters, aromatic rings are present in the derived liquid oil.

### Characterization of liquid fraction by GC–MS analysis

**Figure 6:**
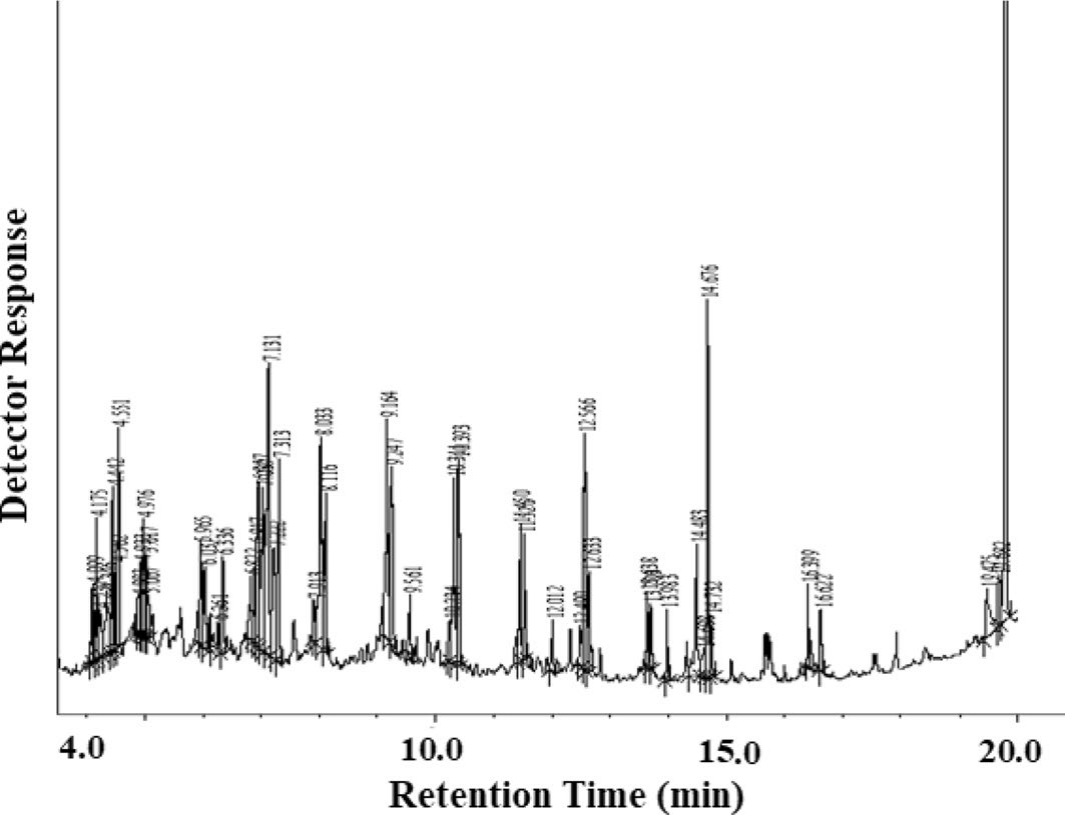
GC–MS Chromatogram of thermally degraded yield (Liquid Oil).

**Figure 7:**
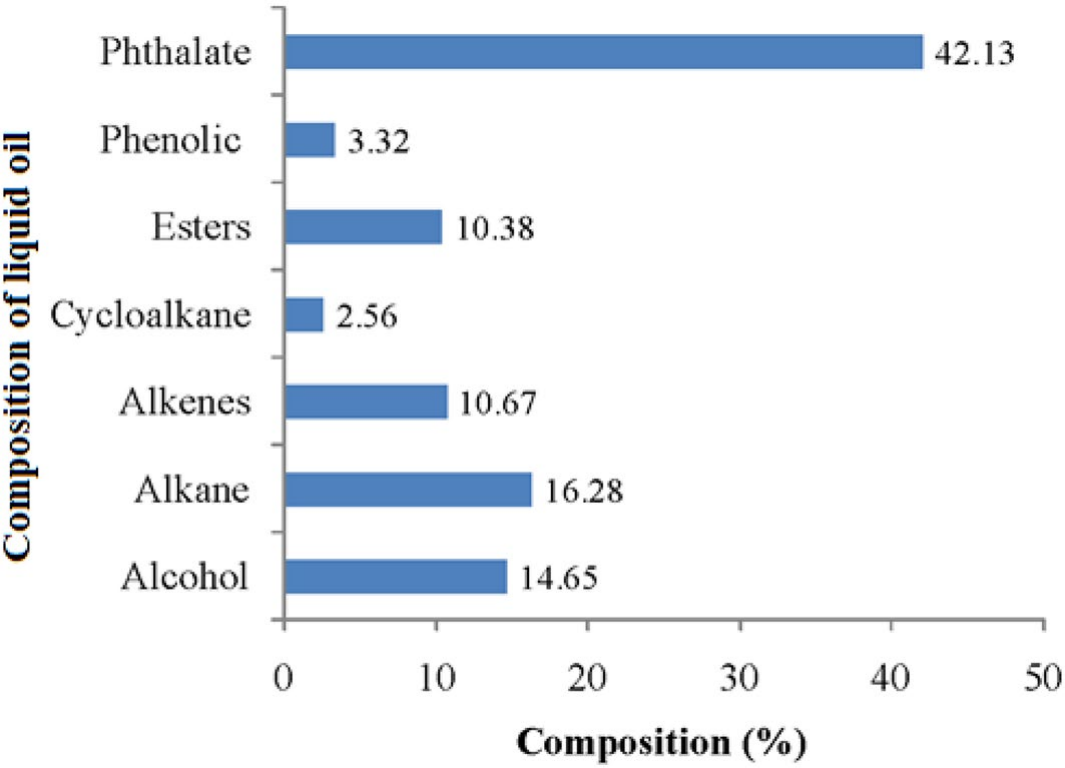
Nature of liquid oil derived from plastic based medical wastes identified by GC–MS analysis.

The GC–MS analysis of the produced liquid fuel was researched to comprehend the nature and kind of the composites in thermally degraded liquid oil. The Total Ion Chromatogram (TIC) of the liquid oil from GC–MS study is shown in Fig. 6. The GC–MS data shows a list of different organic compounds having the functional groups quite similar to FTIR shown in Table 2. According to the TIC peak area, the amount of the types of compounds in percentage is presented in Fig. 7. It was perceived that the thermally degraded liquid oil consisted of aliphatic compounds, mainly of alkane (16.28%), alcohol (14.65%), alkene (10.67%), esters (10.38%), cycloalkane (2.56%) and phenolic compounds (3.32%). The predominant form of major compounds was identified as phthalate group (42.13%) which is widely used as plasticizer in plastic materials.

**Table 2:** Identification of the composition of thermally degraded products by GC–MS.

Types of composition	Name of constituents	Chemical formula	Boiling point (°C)	GC–MS Data	
				Constituent (%, peak area)	Retention time
Alkane	3,3 dimethyl Octane	C_10_H_22_	161	2.54	4.342
	Dodecane	C_12_H_26_	216	0.71	6.037
	Tridecane	C_13_H_28_	234	2.53	7.039
	Tetradecane	C_14_H_30_	255	2.22	8.117
	Pentadecane	C_15_H_32_	270	2.47	9.247
	Heptadecane	C_17_H_36_	302	1.41	11.526
	Eicosane	C_20_H_42_	343	0.77	15.719
	Heneicosane	C_21_H_44_	356	1.36	12.633, 13.703
	Hexacosane	C_26_H_54_	412	2.27	10.393
Cycloalkane	4-cyclohexylDecane	C_16_H_32_	–	1.57	6.917
	1,1 (1,4-butandiyl) bis-Cyclopentane	C_14_H_26_	–	0.99	7.908
Alkene	1-Dodecene	C_12_H_24_	213	0.57	5.965
	1-Tridecene	C_13_H_26_	233	1.35	6.958
	1-Tetradecene	C_14_H_28_	251	2.93	8.033
	1-Pentadecene	C_15_H_30_	268	4.48	9.164, 10.311
	1-Nonadecene	C_19_H_38_	–	0.82	13.642
	7-methyl-1-Undecene	C_12_H_24_	–	0.52	4.933
Alcohol	2-Ethyl-1-hexanol	C_8_H_18_O	184	3.57	4.55, 4.442, 4.502
	1-Heptanol, 2,4-diethyl-	C_11_H_24_O	–	2.12	7.225
	11-Methyldodecanol	C_13_H_28_O	268	7.6	7.133, 7.313
	n-Pentadecanol	C_15_H_32_O	270	1.36	11.45
Ester	Hexadecanoic acid, ethyl ester	C_18_H_36_O_2_	377	3.71	14.675
	Ethyl 14-methyl-hexadecanoate	C_18_H_36_O_2_	–	2.23	12.566, 16.622
	Ethyl 9-hexadecenoate	C_18_H_34_O_2_	356	1.41	14.483
	(E)-9-Octadecenoic acid ethyl ester	C_20_H_38_O_2_	217	0.9	16.399
	Isodecyl methacrylate	C_14_H_26_O_2_	120	0.86	4.975
	Hexadecanoic acid, methyl ester	C_17_H_34_O_2_	417	0.73	13.983
Phenol	Phenol	C_6_H_6_O	182	1.89	4.175, 4.099
	p-Cumenol	C_9_H_12_O	–	1.43	6.117, 6.336
Phthalate	1,3-Benzene dicarboxylic acid, bis(2-ethylhexyl) ester	C_24_H_38_O_4_	400	40.02	19.792
	Bis(2-ethylhexyl) phthalate	C_24_H_38_O_4_	385	2.11	19.683, 19.475

The chemicals found within alkanes, alkenes and cycloalkane groups have high carbon content (C_10_ to C_26_) and these are possibly the degraded products from the mixed polymeric medical wastes. Some other researchers^37,43^ also found similar groups of organic compounds having high carbon contents. The chemical constituent of phthalate group was found as dioctyl isophthalate (DOIP) and diethyl hexyl phthalate (DEHP) which are used as plasticizers in polymeric formulations; especially, in the medical-grade PVC and the typical range of amount used 10–70% (w/w)^44^ for improving the flexibility, durability and stretch ability of the plastics. Shuqi et al.^43^ also found dibutyl phthalate (1.54%) as a plasticizer in the chemical list of GC–MS data in pyrolytic yield. The boiling points of these plasticizers are high (658–673 K) and thus it is expected that such chemicals are not degradable product under the condition applied in this investigation. Analogous chemicals of high boiling point have been found in ester groups which are almost fatty acid ester used as a lubricant or slip agent to reduce the surface coefficient of friction of plastic materials^33,44^. The chemical constituents, found in alcohol group, are used as precursor for the synthesis of DEHP and so it is presumed that the chemicals within ester and alcohol groups are not the thermally degraded product^41^. A small portion of phenol (1.89%) was found in the liquid oil which may produce during thermal degradation of plastics under oxidizing environment^20^. It is concluded from the GC–MS data analysis that the mixed plastic wastes from medical source, which are made from mostly of engineered polymeric resins, are not degraded fully through thermal cracking under oxidizing conditions at a maximum temperature 773 K and various chemical additives used in plastic materials are released in the thermally degraded liquid oil product and there may have environmental effect if such liquid oil is burnt as fuel.

### Properties of liquid fraction as fuel

The characteristics of the as prepared liquid oil are compared to some commercial fuel oils like gasoline and diesel as shown in Table 3. The viscosity of the liquid product (4.0 cSt) is higher than that of diesel (2.8 cSt) and lower than gasoline (6.2 cSt). The density (840 kg/m^3^), flash point (312 K) and gross calorific value (GCV) (41.32 MJ/kg) of the liquid oil are almost similar to liquid fuels reported elsewhere^45^. This liquid fraction can be utilized by blending with commercial fuel oil or this can be further refined to improve the fuel properties.

**Table 3:** Comparison of fuel properties of derived liquid oil with commercial fuels.

Fuel properties	Density (kg/m^3^)	Gross calorific value (MJ/kg)	Flash point (°C)	Viscosity (cSt)	References
Liquid fuel oil	840	41.32	39	4.0	Present work
Gasoline	720–780	42–46	43	6.2	Dash et al.^26^
Diesel	820–850	42–45	53–80	2.8	Rahman et al.^31^
Bio-diesel	880	37–40	100–170	–	Rahman et al.^31^
Heavy fuel oil	940–980	40	90–180	–	Sumi et al.^32^
Kerosene	780–810	43.1–46.2	37–65	–	Roy et al.^33^

### Merits and demerits of thermal cracking of medical based plastic wastes

In a nutshell, the application of the thermal cracking process in the management of medically-based plastic wastes is useful in recovering fuel energy. It has been proved that the procedure breaks down the mixed engineering plastics-based disposable medical supplies having polymers or long-chain hydrocarbons and complex organic molecules as additives. In contrast, it produces a portion of light hydrocarbons alkane and alkene products lower to medium boiling point hydrocarbons, which are useful as a substitute for fossil fuel. Besides, the non-degraded plastic additives that remain in the liquid fraction have a strong influence on fuel properties. The advantage of a thermal cracking process of medical waste is that the batch reactor and associated equipment to maintain the temperature range 723–773 K can be easily commissioned using locally available materials comparatively instead of the high-cost incinerator.

The liquid product comprises a composite mixture of various organic components as described in the GC–MS study. The list of constituents from the GC–MS study showed that no harmful polychlorinated dibenzo-p-dioxins, Polychlorinated dibenzofurans, Polychlorinated biphenyls, and any other hazardous organochlorine products were formed in the liquid fraction, though a significant portion of PVC materials was present in the feedstock. So, the oxidative thermal cracking process could be an alternative to an incinerator for the management of medical-based mixed plastic waste. Moreover, the process will be an alternative thermal technique to disinfect hazardous medical-based plastic wastes without any pre-treatment due to apply high temperature reaction condition and simultaneously to recover clean fuel energy.

In addition, the non-condensate, which is mostly composed of syngas (CO, CO_2_ and H_2_) having economic importance due to its high calorific value used in most chemical and polymer industries. The gas is applied for the generation of power and heat. It can be burned directly as fuel or it is used to produce ethanol and hydrogen. Syngas are also used in the Fischer–Tropsch process to produce liquid hydrocarbons.

The disadvantage of the thermal cracking process is the low yield of product due to the oxidizing condition. Besides, there are some non-degraded and high boiling point compounds that remain in the liquid fraction which has human health and environmental impact. The thermal cracking liquids need further treatments, for instance, centrifugation, filtration, hydro-treating, decanting and desulphurization for use as clean fuels.

## Conclusions

Plastic based medical wastes were successfully employed as a potential feedstock of liquid fuel oil by thermal cracking process in oxidizing conditions. In order to recover liquid oil from the medical waste, the feedstock was thermally cracked at a temperature of 773 ± 10 K and the product yield was found to be 52 wt%. The gross calorific value of the derived liquid oil was found to be 41 MJ/kg that is analogous to the commercial fuel oils. The functional groups of the chemical constituents in the derived liquid fuel oil were examined by using FTIR spectroscopy. The spectrum showed strong absorption bands of both stretching and bending vibrations for alkane, alkene, methylene, alcohol and ester groups. The chemical characteristics of the as prepared liquid oil demonstrate the constituents of a composite mixture of degraded and non-degraded organic compounds derived from the medical based mixed plastic wastes. GC–MS study showed that both degraded polymeric components and non-degraded products existed in the derived fuel oil. The chemical constituents of degraded yield were mostly aliphatic compounds that are mainly of alkane (16.28%), alkene (10.67%), and non-degraded yield was classified as alcohol (14.65%) and ester (10.38%) and predominant compound of phthalate (42.02%) group. The degraded fractions are medium to heavy hydrocarbons having carbon numbers C_8_ to C_26_ and the non-degraded constituents were found mostly as phthalate group. The non-degraded constituents present in the liquid oil may release gases into the environment while burning and harmful to human health. In fact, thermal cracking, pyrolysis or gasification techniques are appraised for the preparation of liquid fuel from plastic based medical waste materials; nonetheless, the liquid fuel needs further treatment or separation techniques, for instance, centrifugation, filtration, hydro-treating, decanting, etc. to be used for the clean fuels in the near future. Finally, it can be concluded that the oxidative thermal cracking process could be an alternative technology instead of an incinerator for not only treating hazardous plastic based medical waste but also to produce clean fuel energy.
